# Social determinant of health patterns and mortality outcomes in US adults

**DOI:** 10.1186/s12889-025-24126-9

**Published:** 2025-08-14

**Authors:** Fangyuan Chen, Ryan D. Nipp, Xuesong Han, Zhiyuan Zheng, Tianci Wang, K. Robin Yabroff, Changchuan Jiang

**Affiliations:** 1 School of Medicine, Tsinghua University, Beijing, China; 2 OU Health Stephenson Cancer Center, University of Oklahoma College of Medicine, Oklahoma City, OK USA; 3 Surveillance and Health Equity Science, American Cancer Society, Atlanta, GA USA; 4 Burnett School of Medicine, Texas Christian University, Fort Worth, TX USA; 5 Division of Medical Oncology and Hematology, Department of Internal Medicine, UT Southwestern Medical Center, Dallas, TX USA

**Keywords:** Social determinants of health, National health interview survey, Mortality outcomes, Cancer-specific mortality, Healthcare access

## Abstract

**Background:**

Social Determinants of Health (SDOH) influences healthcare access, especially in patients with chronic diseases. However, SDOHs were often investigated as single variables. Combination patterns and joint effects of multiple SDOHs are much understudied. This study seeks to identify SDOH patterns in the US general population and their influence in all-cause and specific mortality.

**Methods:**

This study included US adults aged 18 to 79 from 2002 to 2018 National Health Interview Survey (NHIS) and NHIS Linked Mortality Files. 12 SDOHs from 5 domains (healthcare access, education and literacy, economic stability, social isolation, neighborhood cohesion) were selected and binarized from the NHIS, including: material, psychological, and behavioral medical financial hardship, delayed care due to transportation and due to non-transportation factors, education, employment, food security, income, housing security, marital status, and neighborhood cohesion. Key outcomes, including all-cause, cancer-specific mortality, and cardiovascular disease-specific death were identified at quarter and year of death.

**Results:**

From the 105,824 younger adults (18–64 years), and the 23,825 older adults (65–79 years), five distinct SDOH patterns were identified: pattern 1 (31%, few barriers); 2 (20%, unmarried); 3 (17%, unemployed); 4 (15%, both unmarried and unemployed); and 5 (16%, with relatively high rate of non-married status, housing insecurity, and material, psychological, or behavioral medical financial hardships). Compared to pattern 1, pattern 4 and 5 had worse prognosis in all mortality outcomes in both age groups, including all-cause mortality, cancer-specific mortality, and cardiovascular disease-specific mortality in both age groups.

**Conclusions:**

In this study, we found that SDOHs could be clustered into five distinct patterns. Patients who were unmarried and unemployed (pattern 4) or with multiple concurrent adverse SDOHs (pattern 5) had poorest key health outcomes. These findings support comprehensive screening for SDOH profiles to understand cumulative influences of SDOHs on quality of life and clinical outcomes of patients with chronic medical conditions.

**Supplementary Information:**

The online version contains supplementary material available at 10.1186/s12889-025-24126-9.

## Background

Social determinants of health (SDOH) are environmental conditions shaping people’s lives in society– including places where people were born, grew, lived, worked, and aged, and their access to resources including power and money [1–5]. Evidence from national datasets has demonstrated associations among various SDOHs and patients’ health service utilization and other clinical outcomes [2, 6, 7]. For example, financial hardship and transportation barriers to care were associated with increased acute care use and heightened mortality risk [8–10], and the quantity of SDOH-related barriers were correlated with patients’ health service utilization and mortality risk [7, 11]. Reports have showed that social disadvantages related to income, education, geography, and hospital accessibility had a 21% increase in mortality among patients with malignant pleural mesothelioma [12], and that cumulative social disadvantages were associated with more than a twofold higher mortality risk among patients with diabetes [13]. Therefore, identifying specific SDOH barriers and their relations to health outcomes is crucial for understanding and narrowing health disparity.

Current research inadequately addresses the complex nature of SDOHs. For example, SDOHs were often classified into five domains (economic stability, education access and quality, health care access and quality, neighborhood and built environment, and social and community context), however SDOH factors within the same or from different domains often co-exist in various combinations. The lack of a clear understanding of SDOH domains limits our comprehension of their role in health disparities and reduces the effectiveness of interventions to improve health outcome targeting individuals’ SDOHs, such as transportation barriers and food insecurity, especially for patients facing multiple concurrent adverse SDOHs [14, 15]. To effectively reduce disparities and the adverse effects of SDOHs, a more holistic approach is needed, including the need to better understand individuals’ SDOH profiles and their group-specific effects on health outcomes.

Clustering analysis is well-suited for identifying patterns within complex epidemiologic datasets involving numerous variables, and thus is a suitable tool for this goal [16]. In the context of SDOHs, this method can reveal which factors commonly co-exist within specific populations, highlighting opportunities for bundled or integrated intervention. In one study, clustering algorithm detected triad of food insecurity, social isolation and impaired housing or utility payments which frequently occur in same group of people who were younger, black, with lower income [17]. In another study, clustering tool extracted four frequently co-occurring patterns from 84 SDHs in children, from which authors found children with high socioeconomic deprivation pattern exhibited more mental health problem and poorer physical health [18].

In this study, we aimed to identify co-occurrence patterns of SDOHs among patients using clustering analysis and to examine how these patterns are associated with individuals’ mortality outcomes. Specifically, we used a nationally representative cohort to perform a cluster analysis of SDOH across various domains in the U.S. In addition, we aimed to examine associations of SDOH patterns with clinical outcomes, including overall mortality, cancer-specific mortality, and cardiovascular disease (CVD)-specific deaths, in the general population and individuals with common chronic medical conditions. By providing a clearer and holistic understanding of SDOHs, findings from this work will help to inform future social risk screening, stratification, and intervention efforts to improve care access and delivery.

## Methods

### Study participants

Adults were identified from the 2002–2018 National Health Interview Survey (NHIS) and NHIS Linked Mortality Files. The NHIS is an annual, cross-sectional, nationally representative in-person survey of the civilian, non-institutionalized population of the United States. The annual response rate of NHIS was approximately 60% of the eligible adults during the study period [19]. The NHIS Linked Mortality files [20] were used to measure vital status for NHIS respondents through December 31, 2019, which provided 1–20 years of follow-up. Quarter and year of death was available for respondents who died during the study period.

We included adults aged 18–79 years who [1] reported data on at least one SDOH variable and [2] had complete linked mortality data with non-missing vital status as of December 31, 2019. We only included adults aged 18–79 years because the NHIS does not provide single year of age for adults aged 80 years or older, but groups them in a single age category, which limits analysis of mortality. To minimize potential reverse causality, we excluded patients who passed away within the first two years of the initial interview from all analyses. We chose the 6 most common chronic medical conditions for subgroup analyses: [1] hypertension [2], lung disease [3], cardiac disease [4], diabetes [5], morbid obesity, and [6] cancer.

### Social determinants of health

To be consistent with the Centers for Medicare & Medicaid Services (CMS) Health Equity Road Map, twelve SDOH indicators were selected from five domains, and each variable was operationalized as a binary indicator for analysis. These included variables from healthcare access domain: material medical financial hardship (having problem paying medical bills), psychological financial hardship (worrying about paying medical bills), behavioral medical financial hardship (delaying medical care because of affordability), transportation barrier to care (delaying medical care because of transportation barrier), non-transportation barrier to care (delaying medical care because of barriers other than transportation); variable from education and literacy domain (education level: highest level of school being less than high school); variables from economic stability domain: employment status (not working for pay at a job or business in past one week at time of survey), food security (not having sufficient access for nutritious food, defined from results of the ten-item questionnaire), income status (total income less than 100% poverty threshold), housing security (very or moderately worrying about paying the rent); variable from social isolation domain: marital status (widowed, divorced, separated, or never married), and variable from neighborhood cohesion domain (not having sufficient neighborhood support, trust, or connectiveness, defined from results of the four-item questionnaire) (Table S1).

### Outcome measurement

All-cause, cancer-specific (ICD-10: C00-C97 for underlying cause of death), and CVD-specific (ICD-10: I00-I99) mortality were identified at each quarter and year of death. CVD-related death was defined as ischemic heart disease (ICD-10: I20-I25), hypertensive heart disease (ICD-10: I11 and I13), heart failure (ICD-10: I50) and cerebrovascular disease (ICD-10: I60-I69) using underlying cause of death [21].

### Covariates

We chose covariates based on previous research examining SDOHs and existing knowledge on risk factors for poor outcomes [22]. Individuals reported their age, sex, race/ethnicity, and health insurance coverage. The number of comorbid conditions was based on a series of questions about history of hypertension, lung disease, cardiac disease, diabetes, morbid obesity, cancer, stroke, kidney disease, liver diseases, and arthritis. With the long time-period included in the study, we accounted for economic and other secular trends with survey era (2000–2004, 2005–2009, 2010–2014, and 2015–2018).

### Clustering analyses

Patients with missing information in any of the SDOH, comorbidity, and mortality variables were excluded from clustering analysis. To assess multicollinearity among the social determinants of health (SDOH) variables, we calculated variance inflation factors (VIFs) using a linear regression model that included all SDOH variables as predictors and an arbitrary constant outcome. All processed SDOHs were categorical, and thus we applied K-Modes clustering with NHIS sample weights, to cluster patients based on 12 SDOHs [23, 24]. This approach generated between 2 and 10 distinct patient groups per iteration. For each K value, we recorded the clustering cost (using the Python package kmodes v0.12.2, random_state = 42) and determined the optimal number of clusters using the elbow method, implemented with the Python package kneed v0.8.5. We then categorized patients into young (18–64 years) and older (≥ 65 years) for Cox regression analysis, respectively.

### Statistical analyses

For the Cox regression analysis, patients with missing covariate data were further excluded. For both younger (18–64 years) and older (≥ 65 years) age groups, we constructed Cox regression models to examine the association between SDOH patterns and each of three mortality outcomes: all-cause mortality (ACM), cancer-specific mortality (CSM), and cardiovascular disease-specific mortality (CVDM). Time-to-event was defined as the duration from the NHIS interview date (baseline) to the date of death or censoring. Individuals were censored at the end of follow-up (December 31, 2019) or at the time of last known vital status if they were lost to follow-up. For cancer- and CVD-specific mortality analyses, deaths due to other causes were treated as censored events. For each outcome, we adjusted covariates including age, sex, race/ethnicity, region, survey era, comorbid illnesses, functional limitation, and health insurance coverage. We repeated this modeling approach within subgroups of participants with specific chronic conditions, including hypertension, lung disease, cardiac disease, diabetes, morbid obesity, and cancer. All analyses used SAS statistical software, version 9.4 (SAS Institute Inc.) and R software (4.1.2). All statistical significance testing was 2-sided at *p* < 0.05. All analyses used survey weights to account for the complex design of the NHIS linked Mortality File and survey nonresponse [25]. Data analyses were performed from April 01, 2023 to April 20, 2024. The NHIS data are de-identified and publicly available. This study was exempt from IRB review by the Human Research Protection Program (HRPP) at the University of Texas Southwestern Medical Center.

## Results

### Demographics and SDOH characteristics

**Fig. 1:**
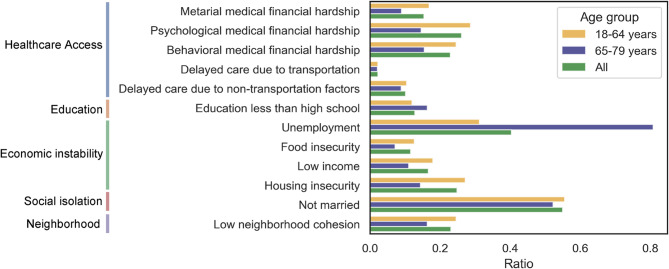
SDOH prevalence across five domains

Of the initial 453,605 entries in the raw NHIS dataset, missing data were observed as follows: material medical financial hardship (220,535; 48.62%), psychological medical financial hardship (58; 0.01%), transportation barriers to care (344; 0.08%), non-transportation barriers to care (336; 0.07%), household income (53,504; 11.80%), housing security (283,997; 62.61%), and mortality status (34,904; 7.69%). After excluding these cases, 130,074 entries remained for the clustering analysis (Fig. 1, Table S2). Delayed care due to transportation had the lowest prevalence in all population (2.2%), consistent in younger (2.2%) or older adults (2.1%). In contrast, being not married had highest prevalence in all (55.0%) and younger adults (55.6%), while unemployment was most prevalent in older adults (80.8%). Compared with older people, the younger adults were more likely to have medical financial hardship (material (16.8% vs. 9.0%), psychological (28.7% vs. 14.6%), or behavioral (24.6% vs. 15.4%)), food insecurity (12.6% vs. 7.1%), housing insecurity (27.1% vs. 14.4%), low income (17.9% vs. 11.0%), and low neighborhood cohesion (24.6% vs. 16.3%). Specifically, VIF value for all SDOH variables were below 1.5, which indicated low multicollinearity among the included variables. Given the small difference between the two groups in all SDOHs (< 15%), we applied clustering on SDOH profiles in all population to derive universal patterns.

### Five distinct SDOH patterns

**Fig. 2:**
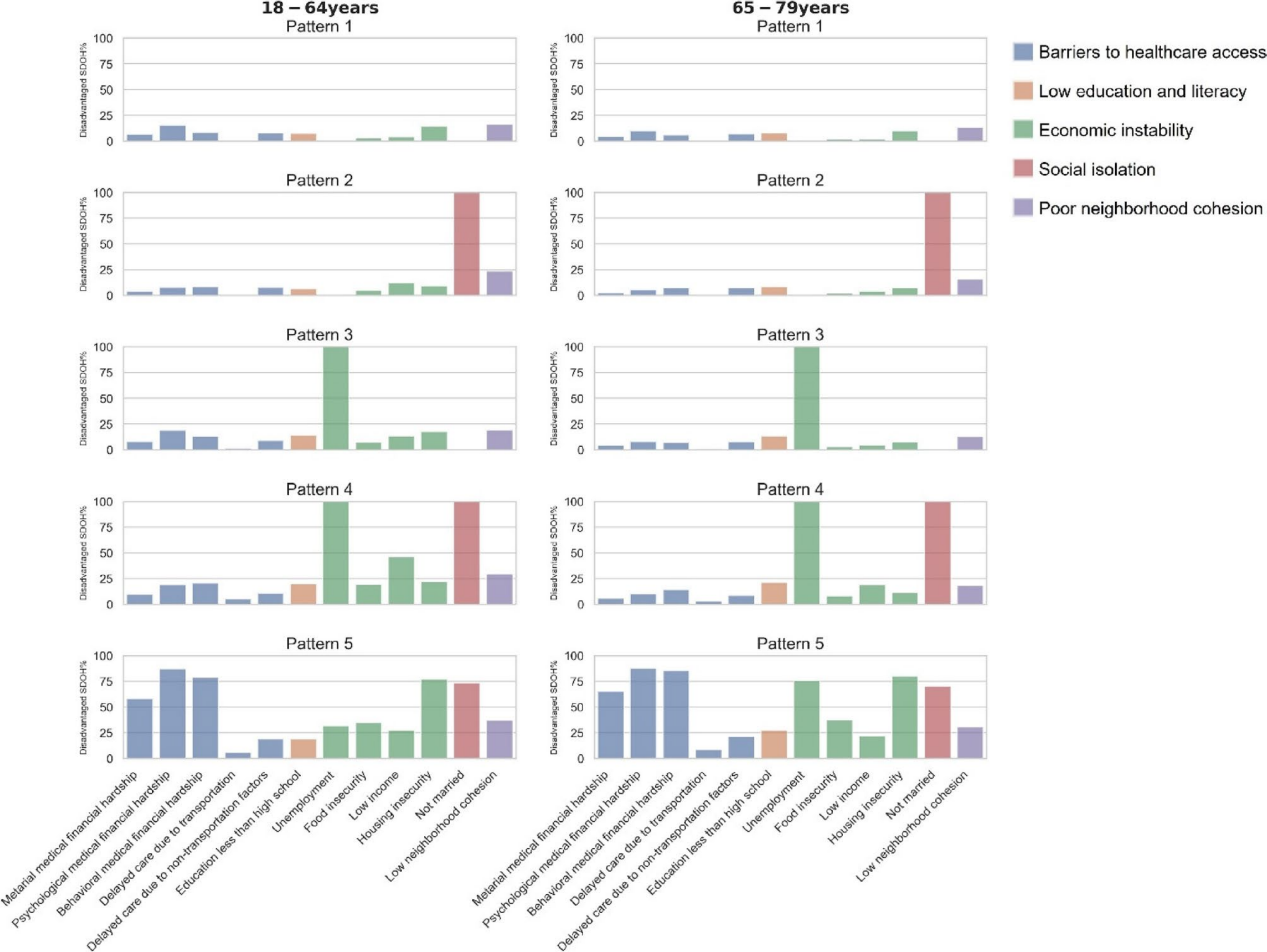
SDOH prevalence of each SDOH pattern across age groups

Five distinct SDOH patterns were identified through universal clustering analysis, with the optimal number of clusters determined using the elbow method (Fig. S1). After applying additional exclusions for missing covariate data (399 entries (0.09%) missing functional limitation data and 1,475 entries (0.33%) missing health insurance data), the final analytic sample included 129,649 adults, comprising 105,824 younger adults (18–64 years) and 23,825 older adults (65–79 years) from this nationally representative dataset (Table 1; Fig. 2). These included: pattern 1 (31%), who had few SDOH barriers; pattern 2 (20%), who were unmarried, and with few other SDOH barriers; pattern 3 (17%), who were unemployed while also with few other SDOH barriers; pattern 4 (15%), who were unmarried and unemployed, and occasionally had other SDOH barriers; and pattern 5 (16%), who frequently had non-married status, housing insecurity, and material, psychological, or behavioral medical financial hardship, while also occasionally had food insecurity, low income, and low neighborhood cohesion. Most patterns were consistent in younger and older adults, though younger participants of pattern 5 were less likely unemployed, and younger participants of pattern 4 were more likely to have low income.

**Table 1:** US adults’ characteristics by age group within 12 months of survey, National health interview survey 2002–2018

Pattern	1 Pattern 1	2 Pattern 2	3 Pattern 3	4 Pattern 4	5 Pattern 5	*p*-value^2^
Number (%)	32,494 (31.2)^*1*^	29,840 (20.3)^*1*^	19,832 (17.0)^*1*^	24,372 (15.0)^*1*^	23,111 (16.4)^*1*^	
Age	45 (36,55)	32 (24,48)	61 (43,69)	45 (23,65)	42 (30,54)	< 0.001
Sex						
Male	57.3	52.2	38.3	44.6	41.8	< 0.001
Female	42.7	47.8	61.7	55.4	58.2	
Race and ethnicity						
White	70.2	63.9	72.8	59.6	54.2	< 0.001
Hispanic	14.4	15.3	13.2	15.3	24.3	
Non-Hispanic Black	7.2	14.9	6.5	19.0	16.8	
Asian and other	8.2	5.9	7.6	6.1	4.7	
Number of health conditions						
0	60.5	70.3	38.8	48.2	46.5	< 0.001
1	23.2	17.8	23.7	19.2	21.6	
2	10.6	7.6	18.5	13.8	14.9	
≥ 3	5.7	4.3	19.0	18.8	17.0	
Any functional limitation						
No	78.3	82.6	53.4	55.0	52.1	< 0.001
Yes	21.7	17.4	46.6	45.0	47.9	
Hypertension						
No	79.8	86.4	62.9	69.2	71.9	< 0.001
Yes	20.2	13.6	37.1	30.8	28.1	
Lung disease						
No	93.0	91.8	89.0	85.7	83.3	< 0.001
Yes	7.0	8.2	11.0	14.3	16.7	
Cardiac disease						
No	93.4	95.0	83.4	86.1	87.4	< 0.001
Yes	6.6	5.0	16.6	13.9	12.6	
Diabetes						
No	94.5	96.0	86.3	87.4	88.4	< 0.001
Yes	5.5	4.0	13.7	12.6	11.6	
Morbid obesity						
No	94.5	95.5	91.6	90.4	88.9	< 0.001
Yes	5.5	4.5	8.4	9.6	11.1	
Cancer						
No	96.2	97.6	89.8	93.1	94.5	< 0.001
Yes	3.8	2.4	10.2	6.9	5.5	
Survey region						
Midwest	23.4	23.8	21.5	21.6	20.8	< 0.001
Northeast	17.9	18.6	17.1	18.8	14.5	
South	35.2	34.3	37.7	37.1	41.1	
West	23.5	23.4	23.7	22.6	23.6	
Survey years						
2010–2014	40.3	39.0	41.0	40.2	45.7	< 0.001
2015–2019	59.7	61.0	59.0	59.8	54.3	
Health insurance coverage						
Age ≤ 64, any private	82.1	74.3	40.5	29.7	39.9	< 0.001
Age ≤ 64, public only	6.1	11.1	13.3	32.3	20.1	
Age ≤ 64, uninsured or missing	6.4	10.5	6.7	11.2	34.2	
Age > 64, Medicare and private	3.2	2.3	18.1	9.0	1.3	
Age > 64, Medicare Advantage/HMO	1.0	0.9	10.1	6.0	1.5	
Age > 64, Medicare and other public	0.0	0.1	1.5	4.0	0.8	
Age > 64, Medicare only/other	1.1	0.9	9.7	7.8	2.1	

*HMO* Health Maintenance Organization, *COPD* Chronic obstructive pulmonary disease

^a^ %

^b^ Chi-squared test with Rao & Scott’s second-order correction

^c^ Other race and ethnicity includes Native American and Alaska Natives, multiple races, and unknown race and/or ethnicity

^d^ Not married includes widowed, divorced, separated, or never married

^e^ Public insurance included Medicare, Medicaid, State Children’s Health Insurance Program, and/or other public hospital/physician coverage. Age ≤ 64 y public insurance comprised people younger than 65 years who had one or more types of public coverage and did not have private coverage. Age ≥ 65 y Medicare + Private, comprised people age 65 and older who had Medicare and private insurance coverage, and did not have Medicare Advantage or HMO. Age ≥ 65 y Medicare Advantage/HMO, comprised people age 65 and older who had Medicare Advantage or HMO and did not have Medicaid coverage. Age ≥ 65 y Medicare only or other, comprised people age 65 and older who had Medicare only and/or one or more of other types of public coverage except for Medicaid or no coverage

^f^ Functional limitations included any self-reported limitation in walking a quarter of a mile, walking up 10 steps without resting, standing or sitting for 2 h, stooping, reaching up over head, carrying 10 pounds, pushing large objects such as a living room chair, shopping, or visiting friends

^g^ Comorbid illnesses included hypertension, diabetes, coronary artery diseases/heart failure/other general heart conditions, cancer, stroke, chronic obstructive lung diseases, kidney disease, liver diseases, arthritis, and morbid obesity

^I^ cardiac disease included coronary artery diseases/heart failure/other general heart conditions

For demographics, pattern 2 individuals were younger (median age of 32 years) than other patterns (median age of 42–61 years). A higher proportion of females were in the pattern 3 (61.7%), 4 (55.4%), and 5 (58.2%) groups, compared with pattern 1 (42.7%) or 2 (47.8%). Notably, pattern 5 and 4 had the highest rate of racial minorities in compared to pattern 1, 2 and 3 (45.8% and 40.4% vs. 27.2–36.1%). Pattern 3, 4, and 5 also had higher proportion of adults with functional limitations (45-47.9% vs. 17.4–21.7%) and comorbid health conditions (51.8–61.2% vs. 29.7–39.5% with ≥ 1 health conditions) than pattern 1 or 2, including hypertension (28.1–37.1% vs. 13.6–20.2%), lung disease (11.0-16.7% vs. 7.0-8.2%), cardiac disease (12.6–16.6% vs. 5.0-6.6%), diabetes (11.6–13.7% vs. 4.0-5.5%), morbid obesity (8.4–11.1% vs. 4.5–5.5%), and cancer (5.5–10.2% vs. 2.4–3.8%). For insurance, pattern 5 were most likely uninsured or with missing data under 64 years (34.2%), compared to other groups (6.4–11.2%).

### SDOH patterns and mortality outcomes

**Fig. 3:**
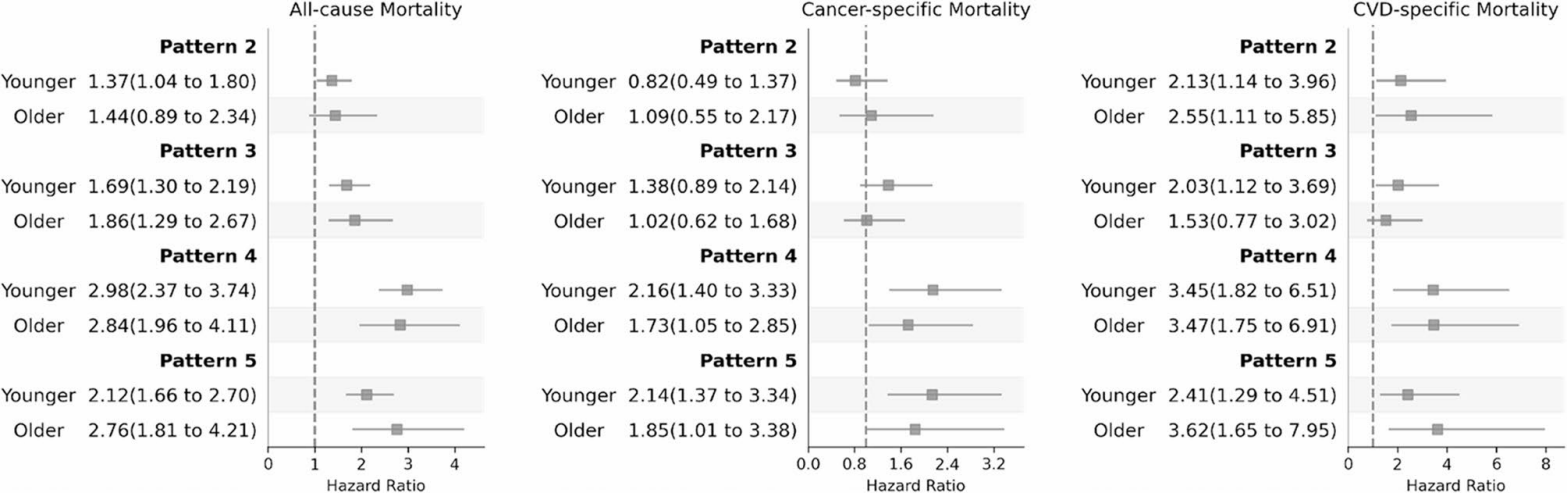
Associations of SDOH patterns with mortality outcomes by age groups

During the study period, 0.97% of the younger adults and 6.47% of the older adults died, respectively. With fully adjusted model (model 4), compared with pattern 1, pattern 2 had higher all-cause mortality in younger adults (HR 1.37, 95% CI 1.04–1.80), and higher CVD-specific mortality in both younger (HR 2.13, 95% CI 1.14–3.96) and older adults (HR 2.55, 95% CI 1.11–5.85). Pattern 3 had increased all-cause mortality in both younger (HR 1.69, 95% CI 1.30–2.19) and older adults (HR 1.86, 95% CI 1.29–2.67), and increased CVD-specific mortality in younger adults (HR 2.03, 95% CI 1.12–3.69). Both pattern 4 and 5 showed poorer prognosis in all mortality outcomes in both age groups, including all-cause mortality (pattern 4: younger, HR 2.98, 95% CI 2.37–3.74; older, HR 2.84, 95% CI 1.96–4.11; pattern 5: younger, HR 2.12, 95% CI 1.66–2.70; older, HR 2.76, 95% CI 1.81–4.21), cancer-specific mortality (pattern 4: younger, HR 2.16, 95% CI 1.40–3.33; older, HR 1.73, 95% CI 1.05–2.85; pattern 5: younger, HR 2.14, 95% CI 1.37–3.34; older, HR 1.85, 95% CI 1.01–3.38), and CVD-specific mortality (pattern 4: younger, HR 3.45, 95% CI 1.82–6.51; older, HR 3.47, 95% CI 1.75–6.91; pattern 5: younger, HR 2.41, 95% CI 1.29–4.51; older, HR 3.62, 95% CI 1.65–7.95) (Fig. 3).

### Sensitivity analyses

Our sensitivity analysis across various health conditions showed similar SDOH clustering patterns in younger and older adults with the most common comorbid health conditions, including hypertension, lung disease, cardiac disease, diabetes, morbid obesity, and cancer (Fig. S2-7). Associations between SDOH patterns and mortality outcomes were also largely similar in sensitivity analyses of people with each comorbid health condition, compared to results from all population. (Fig. S8-13).

## Discussion

In this large, nationally representative study, we used K-Modes clustering of 12 SDOHs across 5 domains and identified distinct patterns associated with unique patient subgroups to 3 mortality outcomes. K-modes clustering is a powerful, unsupervised machine-learning (ML) method for identifying meaningful patterns of participant characteristics in large, multi-variable datasets. As a data-driven method, it highlights the most distinct and impactful feature combinations, providing an objective basis for selecting key variables of interest. Much like principal component analysis (PCA), it reduces the number of input variables via grouping features with similar distribution. This reduction minimizes issues of collinearity and the burden of manual feature selection, while enabling rapid, comprehensive profiling of unique population subgroups. Such machine learning methods were increasingly applied in epidemiological research. For example, a recent U.S.-wide study used such approaches to extract SDOH clusters linked to healthcare disparities, revealing that healthcare infrastructure and access were the primary drivers of cluster composition [26]. Similarly, another study combined Principal Component Analysis and Greedy Equivalence Search algorithms to explore causal pathways among SDOHs, which uncovered a highly interconnected structure where financial stability emerged as a foundational determinant [27].

From the clustering analysis, social isolation and unemployment were the most prevalent individual SDOH components, and their co-existence was significantly associated with increased mortality risk. Additionally, the co-presence of adverse SDOHs, such as housing and food insecurity, alongside transportation barriers, were associated with significantly higher mortality risks. Collectively, our findings support the need for a holistic approach to effectively address these health disparities, including a comprehensive approach to SDOH screening, risk stratification, and targeted interventions to achieve the CDC’s Healthy People 2030 goals [1, 4].

Our study suggests that individual SDOHs are not randomly distributed among patients but form distinct, meaningful patterns that influence health outcomes. This insight aligns with previous work showing overlap between several SDOHs, such as housing and food insecurity [28, 29]. By using unsupervised clustering analysis, our study used data-driven methods (K-Modes clustering) to identify the hidden pattern behind the data and provided a comprehensive, objective evaluation of SDOH distribution across the general population and patients with chronic medical conditions. Notably, these SDOH clustering patterns were highly robust and consistent across age group or comorbid conditions.

Understanding the clustering phenomena of SDOHs is essential for developing effective screening and intervention strategies. While evidence suggests that individual SDOHs, such as food and transportation insecurity, significantly influence patients’ health service usage and outcomes, interventions targeting single SDOHs often fall short [14, 15, 30, 31]. Randomized controlled trials have demonstrated that targeting just one barrier rarely leads to meaningful improvements in patient outcomes [1, 15]. Plausibly, these findings to date may be related to investigators failing to consider clustering phenomenon and/or address concurrent barriers to care. To truly bridge gaps in care access and improve health outcomes, future efforts should adopt a holistic approach, providing comprehensive SDOH evaluation and tailored patient navigation [32]. This integrated strategy will ensure more effective and sustainable health interventions by addressing the full spectrum of social risks [33].

Importantly, social determinants of health (SDOHs) do not contribute equally to individual health outcomes. While prior studies often quantified SDOHs by count (original four citations), this approach overlooks the nuanced effects of specific combinations. In our study, we identified a distinct ‘double-hit’ pattern: the co-occurrence of unemployment and social isolation, which was associated with significantly worse health outcomes than either factor alone. Unemployment has been linked to increased risk of early death through mechanisms such as heightened stress, depression, unhealthy behaviors, reduced healthcare access, and loss of social network [34]. Similarly, not being married (single, divorced, and widowed), as a proxy for social isolation, has been associated with higher all-cause and CVD mortality in men, potentially due to diminished social support, increased loneliness, and adverse health behaviors [35, 36]. The combination of unemployment and social isolation compounds mortality risk, as marriage can buffer the harms of joblessness, and employment can offset risks tied to being unmarried [37]. However, the prevalence and impact of this combination have been understudied. In our analysis, it emerged as a data-driven finding with a notable 17% prevalence and showed that mortality risk as high as groups facing multiple SDOH burdens - highlighting that unemployment and social isolation together should be a top priority for intervention.

On a policy level, recognizing the interactions and cumulative influence of SDOHs is crucial for understanding health disparities in access, treatment, and outcomes. The Centers for Medicare & Medicaid Services (CMS), in alignment with these findings, has strategically emphasized SDOHs in its Health Equity Roadmap [1, 2]. This initiative advocates for the systematic screening and documentation of SDOHs in clinical settings to enhance our understanding of how these social factors influence patient health outcomes and to mitigate related health disparities. To bridge the health gap caused by social risks, a value-based payment system should adjust payments based on patients’ social risk, reflecting hospitals’ and physicians’ efforts to screen, intervene, and educate underserved patients [33, 38–40].

Our study has several limitations meriting discussion. First, although all 12 selected SDOHs across 5 common domains were included, our study is limited by NHIS survey design, which primarily captured individual-level SDOH, without including important community-level or structural determinants such as neighborhood socioeconomic deprivation, environmental exposures, and policy-level factors, and the binary categorization of SDOHs may oversimplify complex dynamics and hide subtle effects. SDOH factors can change dynamically over time; however, in this study, we captured SDOH conditions only at the baseline survey, highlighting the need for future research with longitudinal follow-up and repeated SDOH measurements. Second, reliance on self-reported data raises concerns about recall bias, potentially affecting result accuracy. Some SDOHs, such as transportation barriers, were underreported as survey question didn’t measure the forgone care due to lack of transportation. Statistically, the exclusion of data with missing SDOH data may introduce selection bias. Given individuals with complete SDOH data tend to be healthier with fewer comorbidities, the clustering workflow could result missed populations with poorer health and greater SDOH disadvantages [41]. Additionally, despite the exclusion of individuals who died within two years to mitigate reverse causality, the potential remains that chronic conditions influenced SDOH status over longer periods. Altogether, current limitations underscore the need to enhance data collection and refine analytical methods to accurately evaluate and stratify patients’ social risks based on their SDOH profiles.

## Conclusions

SDOHs may form distinct patterns, which correlate with key health outcomes in US adults with chronic medical conditions. Findings support the need for research to determine effective strategies for comprehensive screening of SDOH profiles to better understand the cumulative influence of SDOHs on quality of life and clinical outcomes in the general population and those with chronic health conditions.

## Supplementary Information

Supplementary Material 1.

## Data Availability

The datasets were derived from sources in the public domain: National Center for Health Statistics, National Health Interview Survey at https://www.cdc.gov/nchs/nhis/documentation/index.html. Dr. Jiang and Dr. Chen had full access to all of the data in the study and take responsibility for the integrity of the data and the accuracy of the data analysis.

## Abbreviations

ACM: All-cause mortality
CSM: Cancer-specific mortality
CVDM: Cardiovascular disease-specific mortality
CDC: Centers for disease control and prevention
SDOH: Social determinants of health
CVD: Cardiovascular disease
NHIS: National health interview survey
CMS: Centers for medicare & medicaid services
FPL: Federal poverty line
