# Supplementary material for: Social determinant of health patterns and mortality outcomes in US adults
Source: BMC Public Health. 2025 Aug 14;25:2761. doi: 10.1186/s12889-025-24126-9 (PMC12351764; doi:10.1186/s12889-025-24126-9)
Supplement: Supplementary file 1 — Supplementary Material 1. [file 12889_2025_24126_MOESM1_ESM.docx]

Table S1. Definition of SDOH variables

| SDOH domain | SDOH variable | Definition |
| --- | --- | --- |
| Healthcare access | Material medical financial hardship (having material medical financial hardship) | Patient answered ‘Yes’ for any of the following questions: 1) In the past 12 months did [fill1: you/anyone in the family] have problems paying or were unable to pay any medical bills? currently have any medical bills that are being paid off over time? (Response category: Yes or No) 2) [fill 1: Do you/Does anyone in your family] currently have any medical bills that are being paid off over time? This could include medical bills being paid off with a credit card, through personal loans, or bill paying arrangements with hospitals or other providers. The bills can be from earlier years as well as this year. |
| Healthcare access | Psychological medical financial hardship (having psychological medical financial hardship) | Patient answered 1 for question: 1) If you get sick or have an accident, how worried are you that you will be able to pay your medical bills? Are you very worried, somewhat worried, or not at all worried? (Response category: 1 Very worried, 2 Somewhat worried, 3 Not at all worried) |
| Healthcare access | Behavioral medical financial hardship (having behavioral medical financial hardship) | Patient answered ‘Yes’ for any of the following questions: 1) Who didn't get needed care? (Anyone else?) (Response category: Yes, No) 2) For which family member was medical care delayed? (Anyone else?) (Response category: Yes, No) 3) DURING THE PAST 12 MONTHS, was there any time when you needed any of the following, but didn't get it because you couldn't afford it? ...Prescription medicines. (Response category: Yes, No) 4) DURING THE PAST 12 MONTHS, was there any time when you needed any of the following, but didn't get it because you couldn't afford it? ...Mental health care or counseling. (Response category: Yes, No) 5) DURING THE PAST 12 MONTHS, was there any time when you needed any of the following, but didn't get it because you couldn't afford it? ...Dental care (including check ups) (Response category: Yes, No) 6) DURING THE PAST 12 MONTHS, was there any time when you needed any of the following, but didn't get it because you couldn't afford it? ...Eyeglasses (Response category: Yes, No) |
| Healthcare access | Transportation barrier to care (delayed care due to transportation) | Patient answered ‘Yes’ for question: There are many reasons people delay getting medical care. Have you delayed getting care for any of the following reasons in the PAST 12 MONTHS? ...You didn't have transportation. (Response category: Yes, No) |
| Healthcare access | Non-transportation barrier to care (delayed care due to non-transportation factors) | Patient answered ‘Yes’ for any of the following questions: 1) There are many reasons people delay getting medical care.  Have you delayed getting care for any of the following reasons in the PAST 12 MONTHS? ...You couldn't get through on the telephone. (Response category: Yes, No) 2) There are many reasons people delay getting medical care. Have you delayed getting care for any of the following reasons in the PAST 12 MONTHS? ...You couldn't get an appointment soon enough. (Response category: Yes or No) 3) There are many reasons people delay getting medical care. Have you delayed getting care for any of the following reasons in the PAST 12 MONTHS? ...Once you get there, you have to wait too long to see the doctor. (Response category: Yes or No) 4) There are many reasons people delay getting medical care. Have you delayed getting care for any of the following reasons in the PAST 12 MONTHS? ...The (clinic/doctor's) office wasn't open when you could get there. (Response category: Yes or No) |
| Education and literacy | Low education level (less than high school) | Patient answered 00-13 for question: What is the HIGHEST level of school [fill: you have/ALIAS has] completed or the highest degree [fill: you have/ALIAS has] received? Please tell me the number from the card. (Response category: 00 Never attended/kindergarten only; 01 1st grade; 02 2nd grade; 03 3rd grade; 04 4th grade; 05 5th grade; 06 6th grade; 07 7th grade; 08 8th grade; 09 9th grade; 10 10th grade; 11 11th grade; 12 12th grade, no diploma; 13 GED or equivalent; 14 High School Graduate; 15 Some college, no degree; 16 Associate degree: occupational, technical, or vocational program; 17 Associate degree: academic program; 18 Bachelor's degree (Example: BA, AB, BS, BBA); 19 Master's degree (Example: MA, MS, MEng, MEd, MBA); 20 Professional School degree (Example: MD, DDS, DVM, JD); 21 Doctoral degree (Example: PhD, EdD); 96 Child under 5 years old |
| Economic stability | Employment (unemployment) | Patient answered 2-5 for question: Corrected employment status last week (Response category: 1 Working for pay at a job or business; 2 With a job or business but not at work; 3 Looking for work; 4 Working, but not for pay, at a family-owned job or business; 5 Not working at a job or business and not looking for work) |
| Economic stability | Food security (food insecurity) | If patient satisfies ≥ 3 following conditions: 1) Answering 1 or 2 for question: The first statement is "[fill 2: I/We] worried whether [fill 3: my/our] food would run out before [fill 4: I/we] got money to buy more." Was that often true, sometimes true, or never true for [fill 1: you/your family] in the last 30 days? (Response category: 1 Often true; 2 Sometimes true; 3 Never true) 2) Answering 1 or 2 for question: "The food that [fill 1: I/we] bought just didn't last, and [fill 1: I/we] didn't have money to get more." Was that often true, sometimes true, or never true for [fill 2: you/your family] in the last 30 days? (Response category: 1 Often true; 2 Sometimes true; 3 Never true) 3) Answering 1 or 2 for question: "[fill 1: I/We] couldn't afford to eat balanced meals." Was that often true, sometimes true, or never true for [fill 2: you/your family] in the last 30 days? (Response category: 1 Often true; 2 Sometimes true; 3 Never true) 4) Answering ‘Yes’ for question: In the last 30 days, did [fill 1: you/you or other adults in your family] ever cut the size of your meals or skip meals because there wasn't enough money for food? (Response category: Yes No) 5) Answering ‘Yes’ for question: In the last 30 days, did you ever eat less than you felt you should because there wasn't enough money for food? (Response category: Yes No) 6) Answering ‘Yes’ for question: In the last 30 days, were you ever hungry but didn't eat because there wasn't enough money for food? (Response category: Yes No)  7) Answering ‘Yes’ for question: In the last 30 days, did you lose weight because there wasn't enough money for food? (Response category: Yes No)  8) Answering ‘Yes’ for question: In the last 30 days, did [fill 1: you/you or other adults in your family] ever not eat for a whole day because there wasn't enough money for food? (Response category: Yes No) 9) Answer ≥ 3 for question: In the last 30 days, how many days did this happen? Adults in the family cut the size of their meals or skipped meals in the last 30 days because there wasn't enough money for food (Response category: 01-30 days) 10) Answer ≥ 3 for question: In the last 30 days, how many days did this happen? All families where the adult(s) did not eat for a whole day, in the last 30 days, because there wasn't enough money for food (Response category: 01-30 days) |
| Economic stability | Income (low income) | Patient answered 1 for question: Was your total [fill1: family/ ] income from all sources less than [fill2: 100% poverty threshold] or [fill2: 100% poverty threshold] or more? (Response category: 1 Less than [fill2: 100% of poverty threshold]; 2 [fill2: 100% poverty threshold] or more) |
| Economic stability | Housing security (housing insecurity) | Patient answered 1 or 2 for question:  How worried are you right now about not being able to pay your rent, mortgage, or other housing costs? Are you… (Response category: 1 Very worried; 2 Moderately worried; 3 Not too worried; 4 Not worried at all) |
| Social isolation | Marital status (not married) | Patient answered 2, 3, 4, or 5 for question:  [fill: Are you/Is ALIAS] now married, widowed, divorced, separated, never married, or living with a partner? (Response category: 1 Married; 2 Widowed; 3 Divorced; 4 Separated; 5 Never Married; 6 Living with partner) |
| Neighborhood cohesion | Neighborhood cohesion (poor neighborhood cohesion) | If sum of answered numbers ≥ 10 for following questions: 1) How much do you agree or disagree with the following statements about your neighborhood? People in this neighborhood help each other out. Would you say… (Response category: 1 Definitely agree; 2 Somewhat agree; 3 Somewhat disagree; 4 Definitely disagree) 2) How much do you agree or disagree with the following statements about your neighborhood? There are people I can count on in this neighborhood. Would you say… (Response category: 1 Definitely agree; 2 Somewhat agree; 3 Somewhat disagree; 4 Definitely disagree)  3) How much do you agree or disagree with the following statements about your neighborhood? People in this neighborhood can be trusted. Would you say… (Response category: 1 Definitely agree; 2 Somewhat agree; 3 Somewhat disagree; 4 Definitely disagree)  4) How much do you agree or disagree with the following statements about your neighborhood? This is a close-knit neighborhood. Would you say… (Response category: 1 Definitely agree; 2 Somewhat agree; 3 Somewhat disagree; 4 Definitely disagree) |

**Table S2. Prevalence of having adverse SDOH in 12 SDOHs across five domains**

| SDOH Domain | SDOH Variable | Age group | Percentage, % |
| --- | --- | --- | --- |
| Healthcare Access | Material medical financial hardship | 18-64 years | 16.8 |
| Healthcare Access | Psychological medical financial hardship | 18-64 years | 28.7 |
| Healthcare Access | Behavioral medical financial hardship | 18-64 years | 24.6 |
| Healthcare Access | Delayed care due to transportation | 18-64 years | 2.2 |
| Healthcare Access | Delayed care due to non-transportation factors | 18-64 years | 10.4 |
| Education and literacy | Education less than high school | 18-64 years | 11.9 |
| Economic instability | Unemployment | 18-64 years | 31.3 |
| Economic instability | Food insecurity | 18-64 years | 12.6 |
| Economic instability | Low income | 18-64 years | 17.9 |
| Economic instability | Housing insecurity | 18-64 years | 27.1 |
| Social isolation | Not married | 18-64 years | 55.6 |
| Neighborhood | Low neighborhood cohesion | 18-64 years | 24.6 |
| Healthcare Access | Material medical financial hardship | 65-79 years | 9.0 |
| Healthcare Access | Psychological medical financial hardship | 65-79 years | 14.6 |
| Healthcare Access | Behavioral medical financial hardship | 65-79 years | 15.4 |
| Healthcare Access | Delayed care due to transportation | 65-79 years | 2.1 |
| Healthcare Access | Delayed care due to non-transportation factors | 65-79 years | 8.9 |
| Education and literacy | Education less than high school | 65-79 years | 16.4 |
| Economic instability | Unemployment | 65-79 years | 80.8 |
| Economic instability | Food insecurity | 65-79 years | 7.1 |
| Economic instability | Low income | 65-79 years | 11.0 |
| Economic instability | Housing insecurity | 65-79 years | 14.4 |
| Social isolation | Not married | 65-79 years | 52.2 |
| Neighborhood | Low neighborhood cohesion | 65-79 years | 16.3 |
| Healthcare Access | Material medical financial hardship | All | 15.4 |
| Healthcare Access | Psychological medical financial hardship | All | 26.1 |
| Healthcare Access | Behavioral medical financial hardship | All | 22.9 |
| Healthcare Access | Delayed care due to transportation | All | 2.2 |
| Healthcare Access | Delayed care due to non-transportation factors | All | 10.1 |
| Education and literacy | Education less than high school | All | 12.8 |
| Economic instability | Unemployment | All | 40.4 |
| Economic instability | Food insecurity | All | 11.6 |
| Economic instability | Low income | All | 16.6 |
| Economic instability | Housing insecurity | All | 24.8 |
| Social isolation | Not married | All | 55.0 |
| Neighborhood | Low neighborhood cohesion | All | 23.1 |

Figure S1. Selecting optimal number of clusters


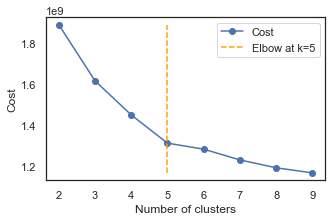


Cost of K-Modes clustering with cluster numbers ranging from 2 to 9. Optimal number of clusters (n=5) chosen via the elbow method.

Figure S2. SDOH patterns in adults with hypertension


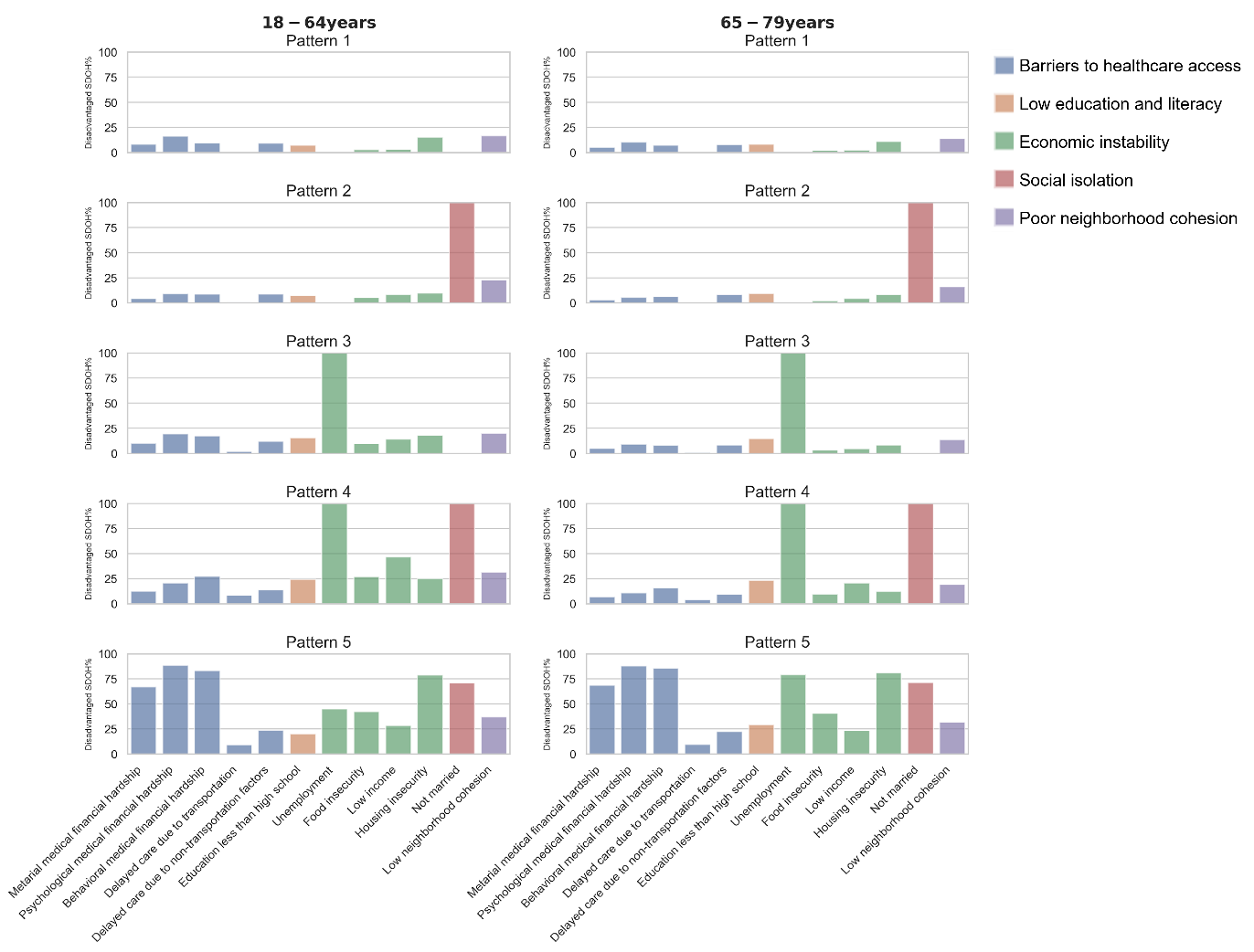


Ratio of having adverse SDOHs in 12 SDOHs in each of the five SDOH patterns in younger (18-64 years) and older adults (65-79 years) with hypertension.

Figure S3. SDOH patterns in adults with lung disease


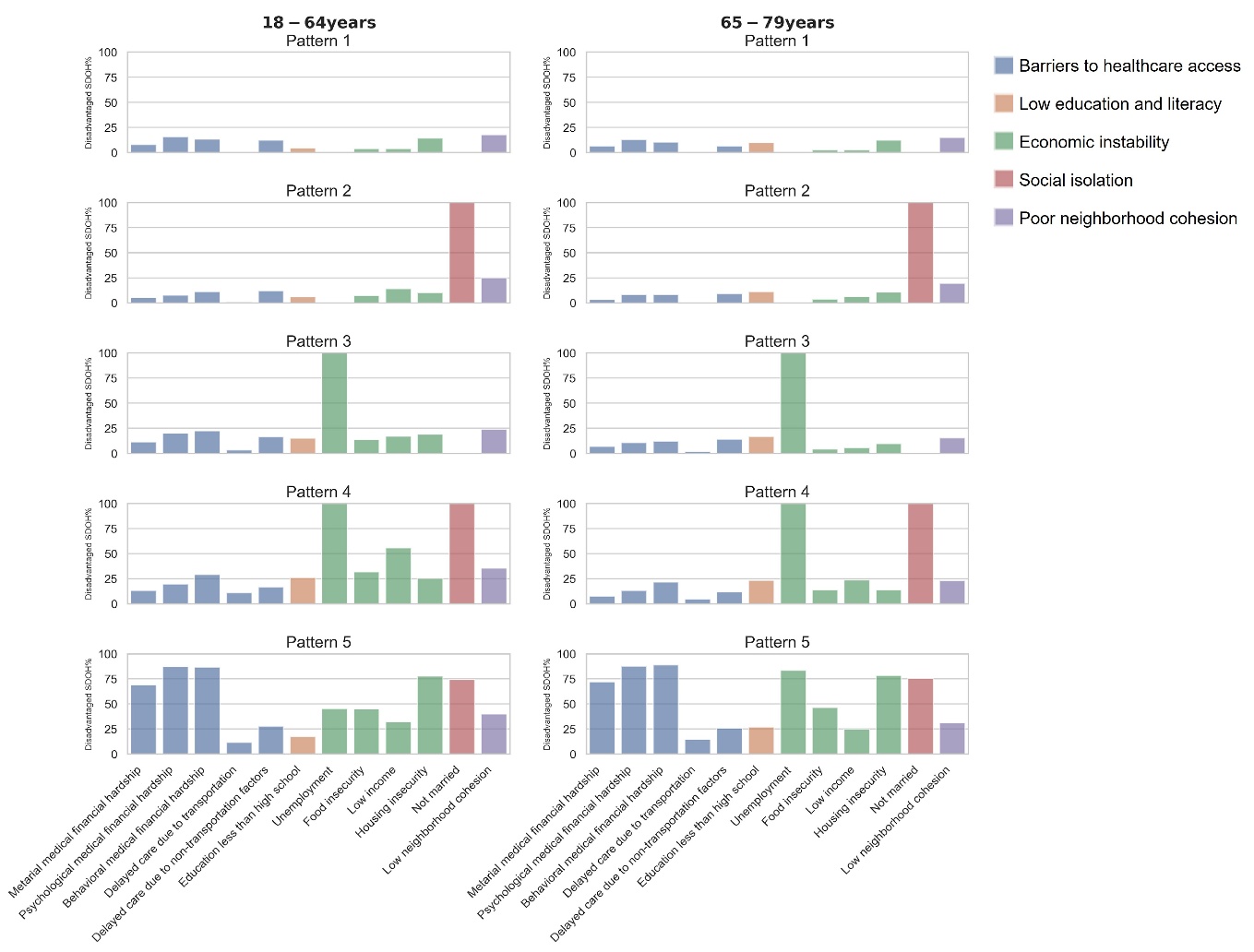


Ratio of having adverse SDOHs in 12 SDOHs in each of the five SDOH patterns in younger (18-64 years) and older adults (65-79 years) with lung disease.

Figure S4. SDOH patterns in adults with cardiac disease


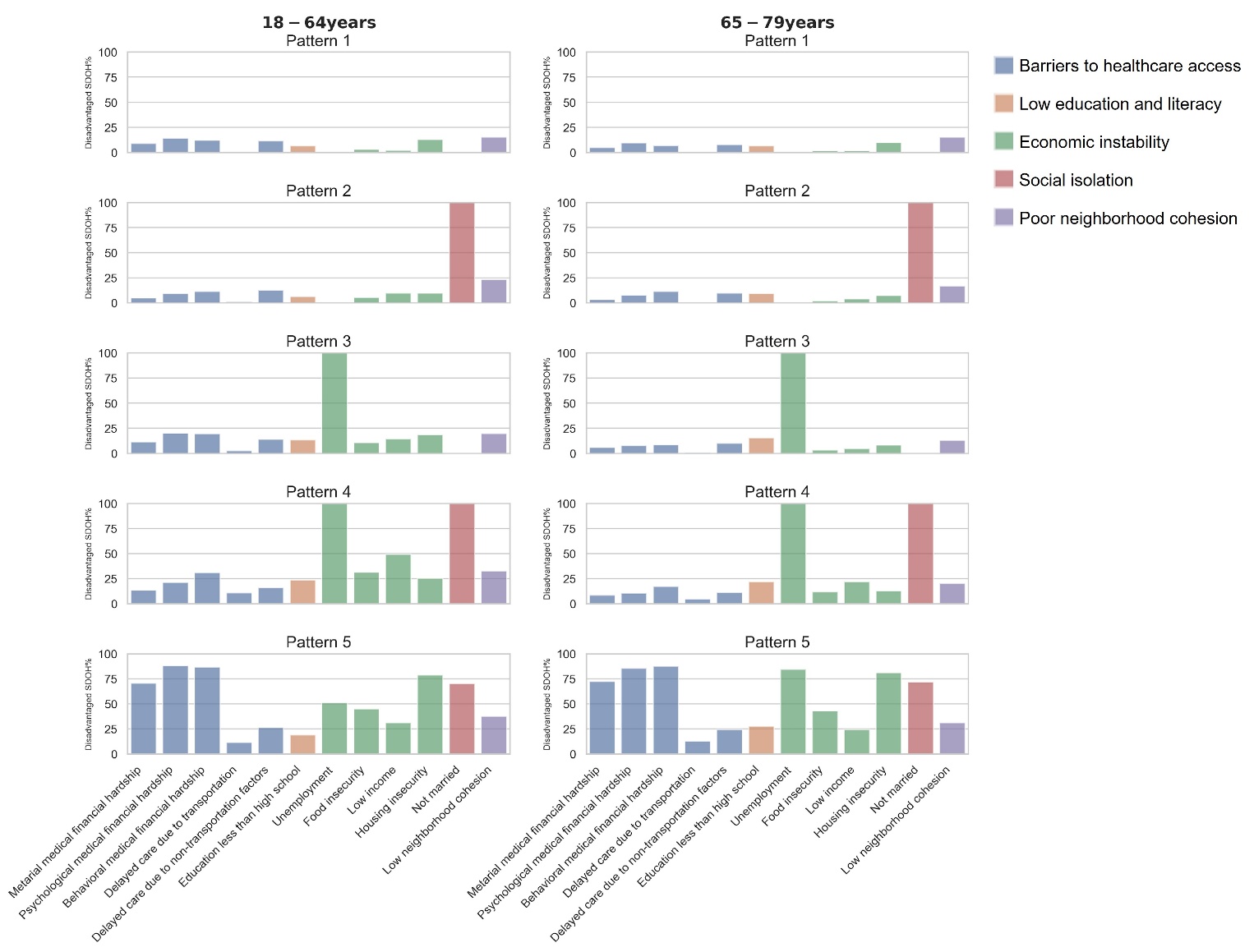


Ratio of having adverse SDOHs in 12 SDOHs in each of the five SDOH patterns in younger (18-64 years) and older adults (65-79 years) with cardiac disease.

Figure S5. SDOH patterns in adults with diabetes


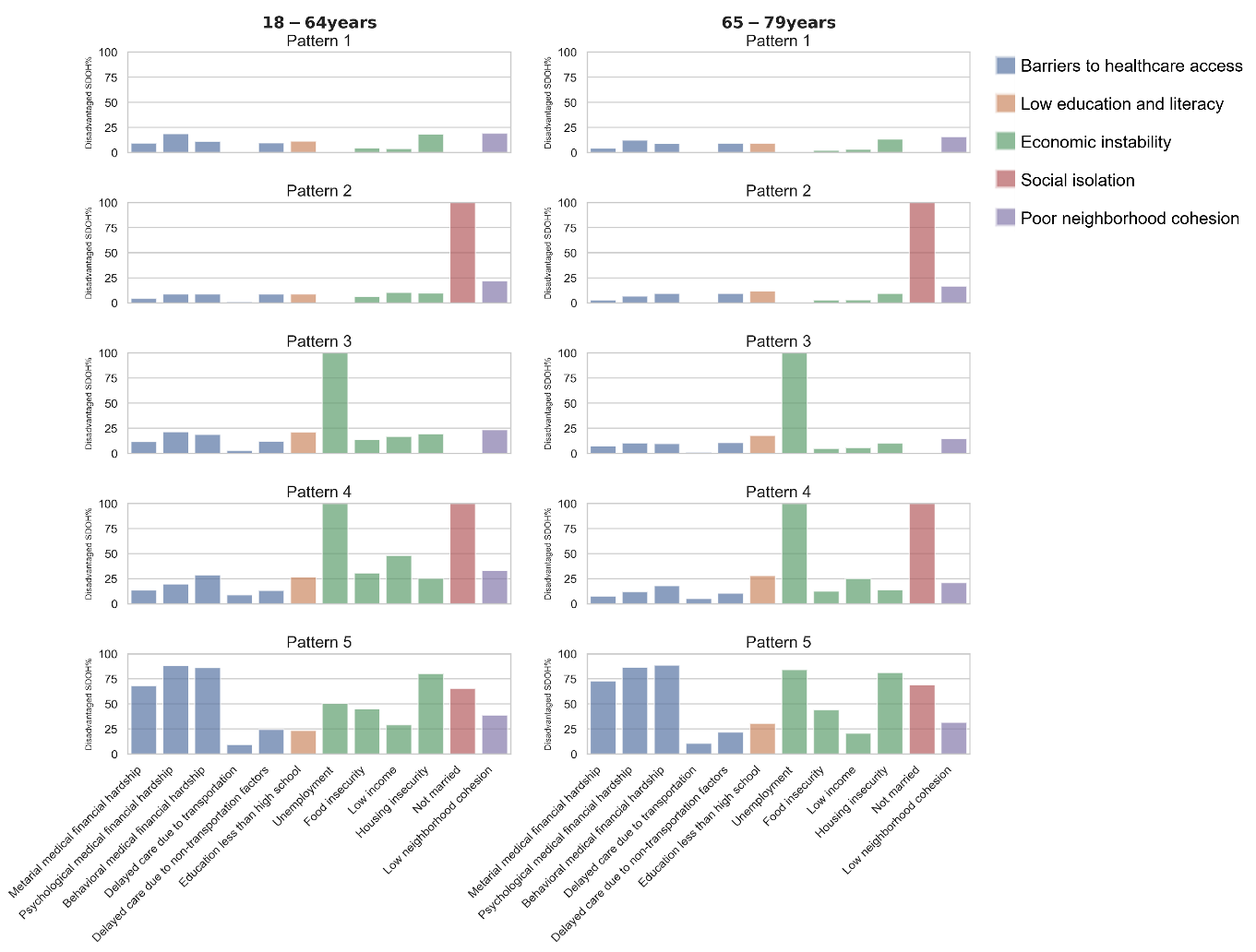


Ratio of having adverse SDOHs in 12 SDOHs in each of the five SDOH patterns in younger (18-64 years) and older adults (65-79 years) with diabetes.

Figure S6. SDOH patterns in adults with morbid obesity


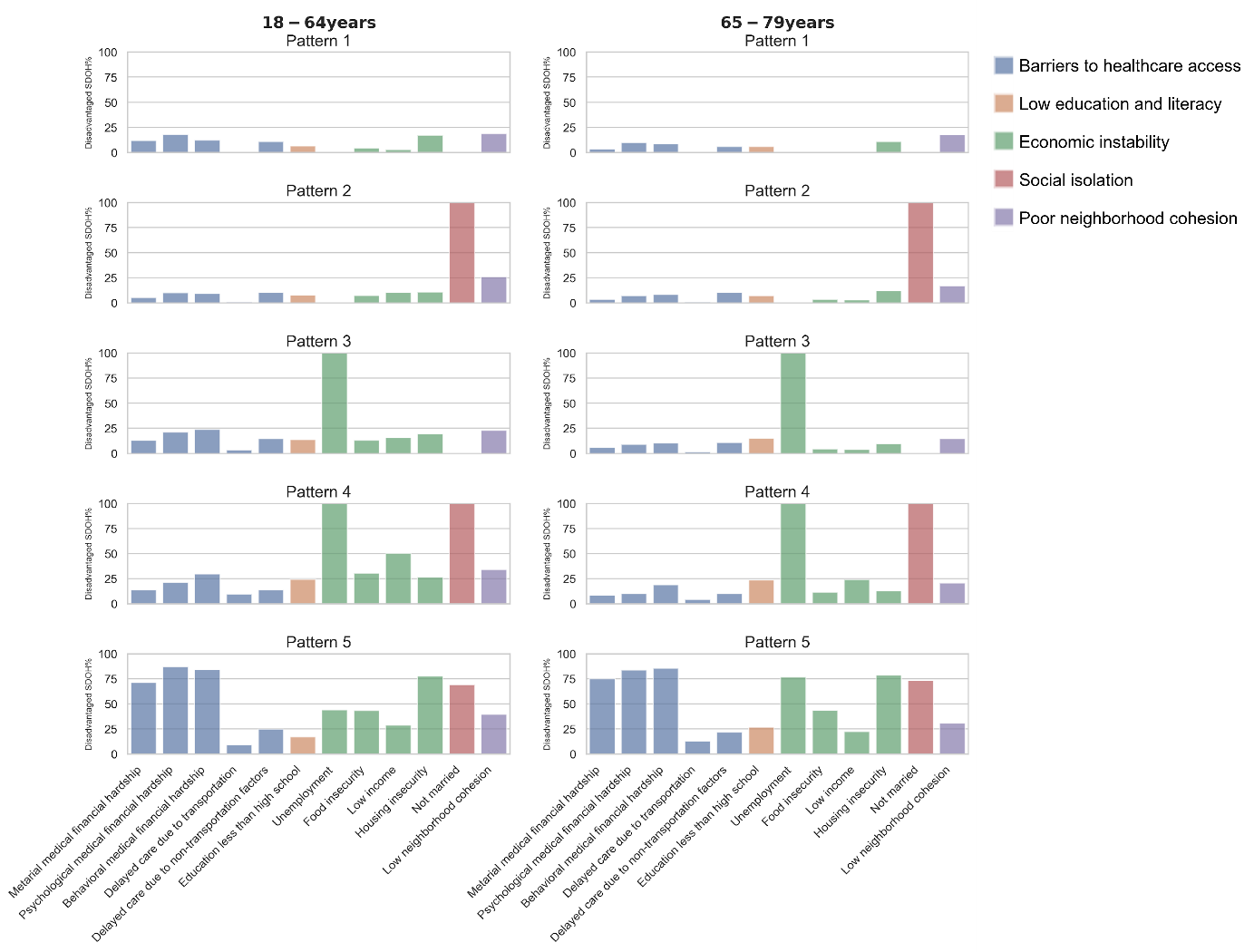


Ratio of having adverse SDOHs in 12 SDOHs in each of the five SDOH patterns in younger (18-64 years) and older adults (65-79 years) with morbid obesity.

Figure S7. SDOH patterns in adults with cancer


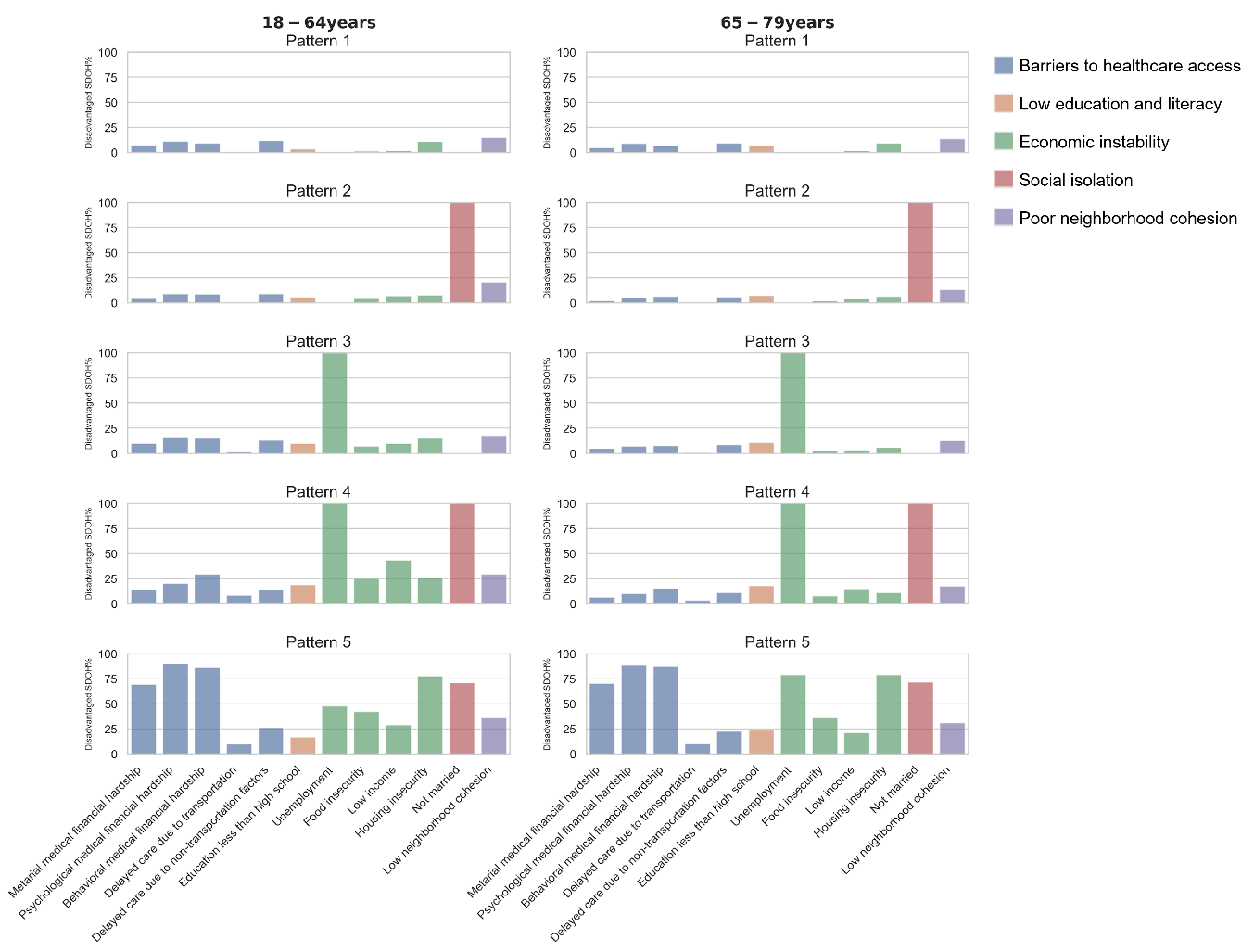


Ratio of having adverse SDOHs in 12 SDOHs in each of the five SDOH patterns in younger (18-64 years) and older adults (65-79 years) with cancer.

Figure S8. SDOH patterns and key health outcomes in adults with hypertension


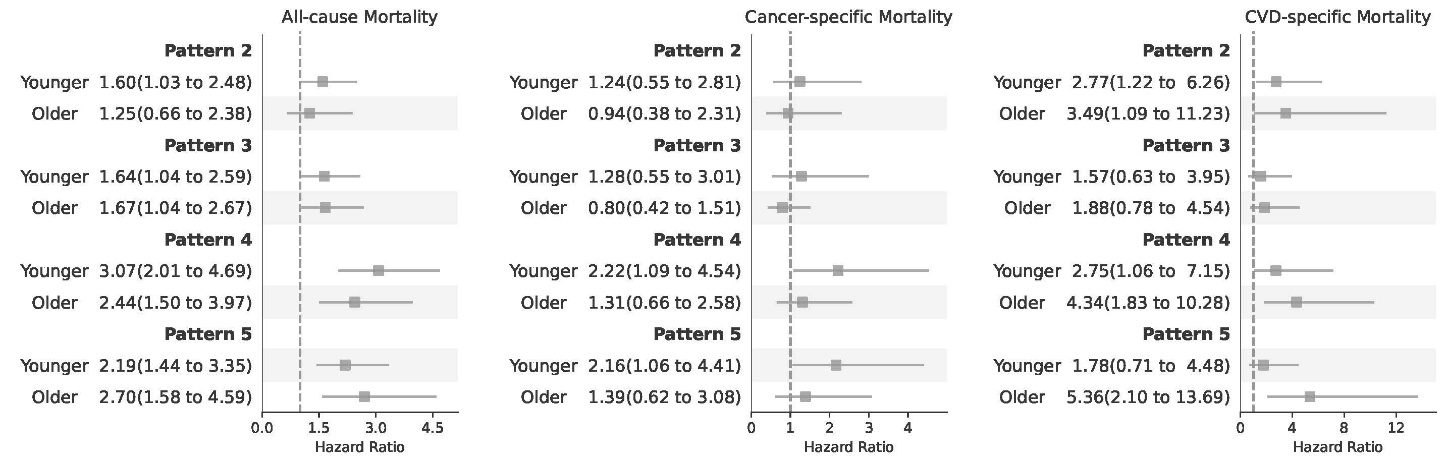


Hazard ratio of SDOH patterns 2-5 (pattern 1 as reference) in all-cause mortality, cancer-specific mortality, and CVD-specific mortality in younger (18-64 years) and older (65-79 years) adults with hypertension, adjusting for age, region, survey era, sex, race, comorbid illnesses, functional limitation, and insurance.

Figure S9. SDOH patterns and key health outcomes in adults with lung disease


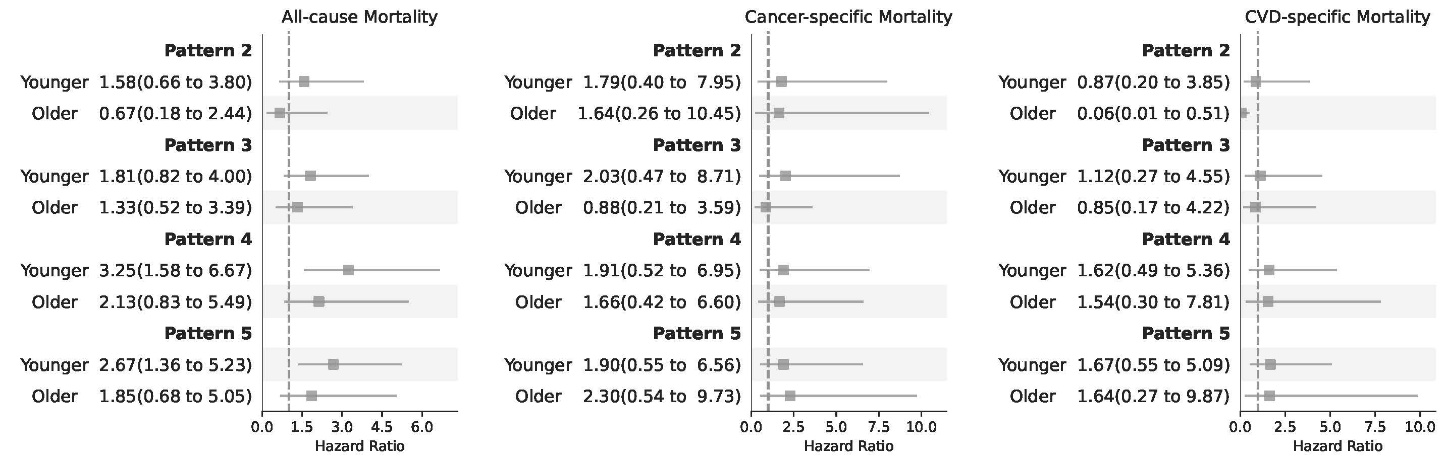


Hazard ratio of SDOH patterns 2-5 (pattern 1 as reference) in all-cause mortality, cancer-specific mortality, and CVD-specific mortality in younger (18-64 years) and older (65-79 years) adults with lung disease, adjusting for age, region, survey era, sex, race, comorbid illnesses, functional limitation, and insurance.

Figure S10. SDOH patterns and key health outcomes in adults with cardiac disease


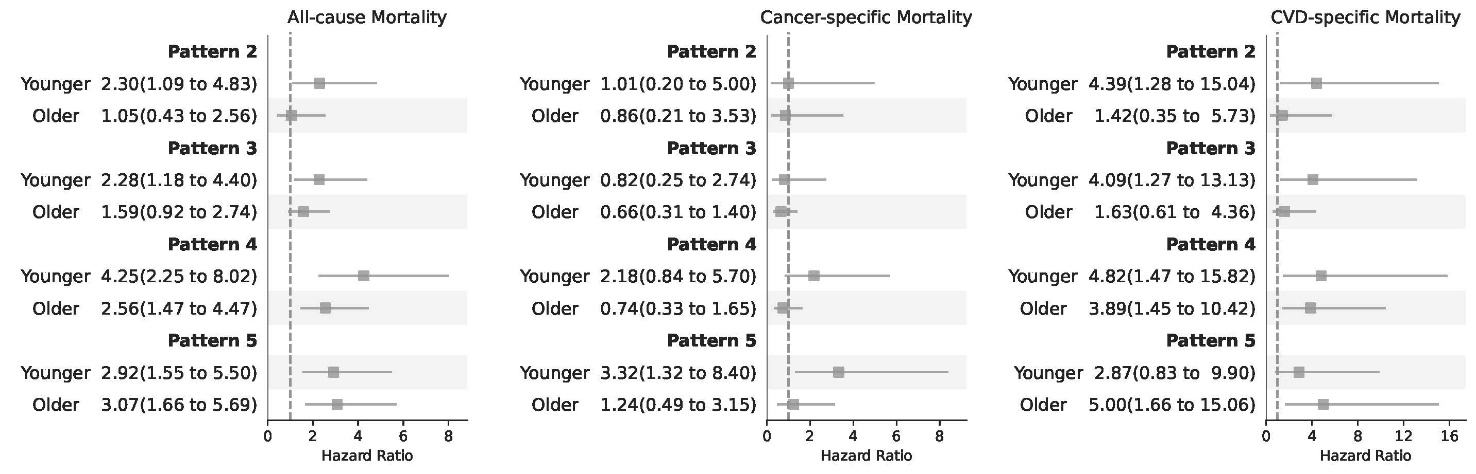


Hazard ratio of SDOH patterns 2-5 (pattern 1 as reference) in all-cause mortality, cancer-specific mortality, and CVD-specific mortality in younger (18-64 years) and older (65-79 years) adults with cardiac disease, adjusting for age, region, survey era, sex, race, comorbid illnesses, functional limitation, and insurance.

Figure S11. SDOH patterns and key health outcomes in adults with diabetes


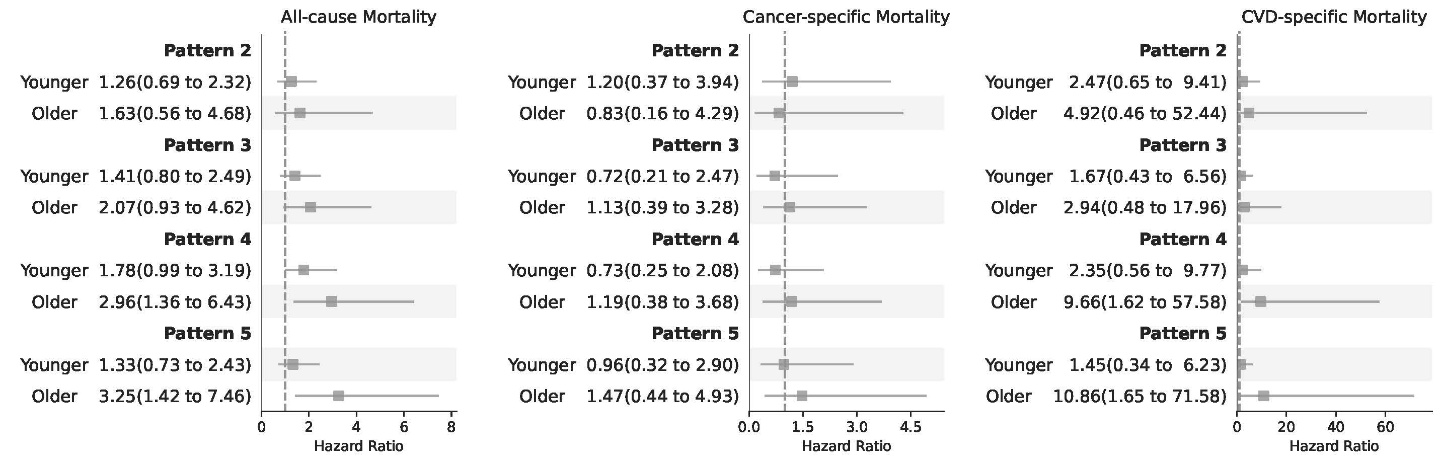


Hazard ratio of SDOH patterns 2-5 (pattern 1 as reference) in all-cause mortality, cancer-specific mortality, and CVD-specific mortality in younger (18-64 years) and older (65-79 years) adults with diabetes, adjusting for age, region, survey era, sex, race, comorbid illnesses, functional limitation, and insurance.

Figure S12. SDOH patterns and key health outcomes in adults with morbid obesity


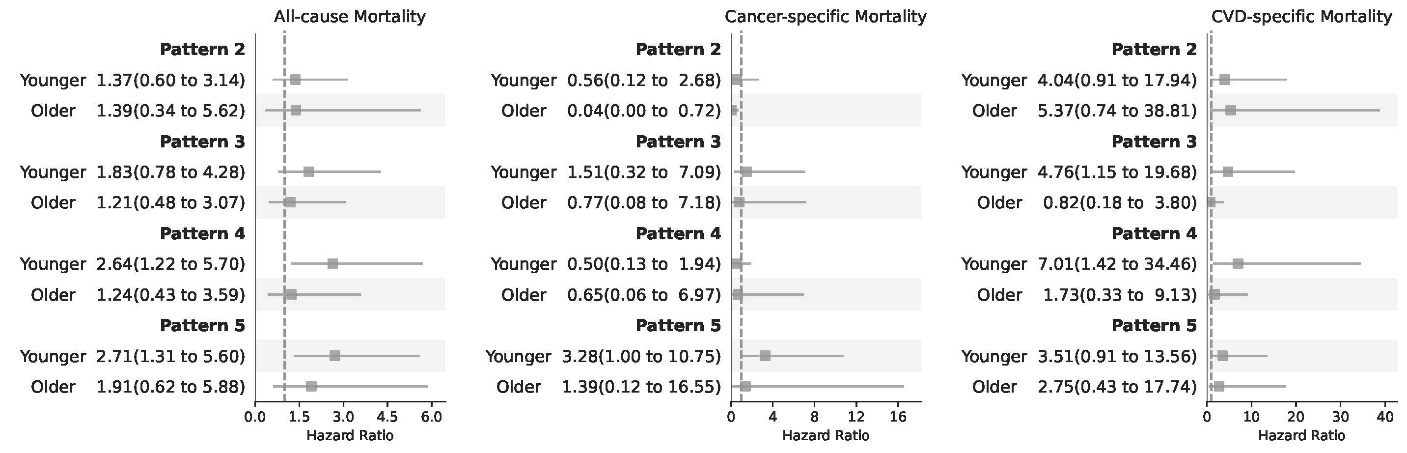


Hazard ratio of SDOH patterns 2-5 (pattern 1 as reference) in all-cause mortality, cancer-specific mortality, and CVD-specific mortality in younger (18-64 years) and older (65-79 years) adults with morbid obesity, adjusting for age, region, survey era, sex, race, comorbid illnesses, functional limitation, and insurance.

Figure S13. SDOH patterns and key health outcomes in adults with cancer


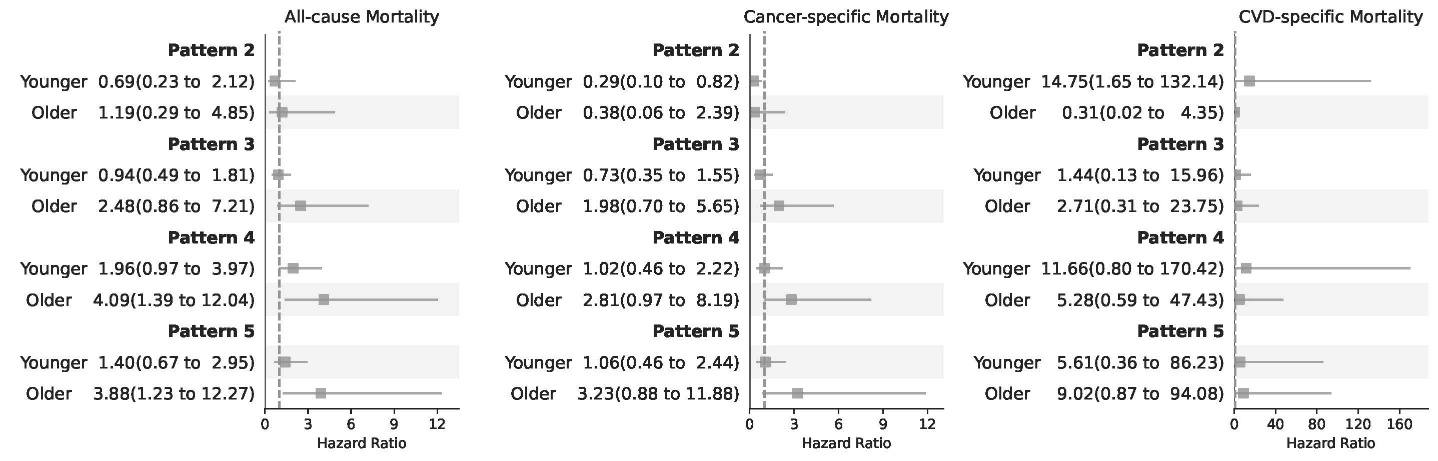


Hazard ratio of SDOH patterns 2-5 (pattern 1 as reference) in all-cause mortality, cancer-specific mortality, and CVD-specific mortality in younger (18-64 years) and older (65-79 years) adults with cancer, adjusting for age, region, survey era, sex, race, comorbid illnesses, functional limitation, and insurance.
